# Self-Assembly Regulated Photocatalysis of Porphyrin-TiO_2_ Nanocomposites

**DOI:** 10.3390/molecules29163872

**Published:** 2024-08-15

**Authors:** Yisheng Liu, Xinpeng Lv, Yong Zhong, Gaoyang Wang, Shuanghong Liu, Sudi Chen, Cai Qi, Mu He, Ping Shangguan, Zhengqun Luo, Xi Li, Jincheng Guo, Jiajie Sun, Feng Bai, Jiefei Wang

**Affiliations:** 1 Academy for Advanced Interdisciplinary Studies, Henan Key Laboratory of Brain Targeted Bio-Nanomedicine, School of Life Sciences, Henan University, Kaifeng 475004, China; 2 Key Laboratory for Special Functional Materials of Ministry of Education, National & Local Joint Engineering Research Center for High-Efficiency Display and Lighting Technology, School of Nanoscience and Materials Engineering, Collaborative Innovation Center of Nano Functional Materials and Applications, Henan University, Kaifeng 475004, China; 3 School of Physics and Electronics, Henan University, Kaifeng 475004, China

**Keywords:** porphyrin, self-assembly, photocatalysis, hydrogen generation

## Abstract

Photoactive artificial nanocatalysts that mimic natural photoenergy systems can yield clean and renewable energy. However, their poor photoabsorption capability and disfavored photogenic electron–hole recombination hinder their production. Herein, we designed two nanocatalysts with various microstructures by combining the tailored self-assembly of the meso-tetra(p-hydroxyphenyl) porphine photosensitizer with the growth of titanium dioxide (TiO_2_). The porphyrin photoabsorption antenna efficiently extended the absorption range of TiO_2_ in the visible region, while anatase TiO_2_ promoted the efficient electron–hole separation of porphyrin. The photo-induced electrons were transferred to the surface of the Pt co-catalyst for the generation of hydrogen via water splitting, and the hole was utilized for the decomposition of methyl orange dye. The hybrid structure showed greatly increased photocatalytic performance compared to the core@shell structure due to massive active sites and increased photo-generated electron output. This controlled assembly regulation provides a new approach for the fabrication of advanced, structure-dependent photocatalysts.

## 1. Introduction

Energy sustainability is crucial for anthropogenic activity and the development of societies [1]. Recent technological advancements, economic development, and population growth have exerted increasing pressure on the global energy supply. However, with the decline of fossil fuel reserves and climate change, the development of green sustainable alternative energy has become a major challenge [2,3]. To address this energy shortage while reducing the dependency on fossil fuels, many new forms of energy, including wind energy, solar energy, geothermal energy, hydropower, hydrogen energy, and bioenergy have been explored [4,5,6,7]. Renewable, sustainable, and green hydrogen energy is considered an attractive alternative to fossil fuels [8,9]. Furthermore, the generation of hydrogen fuel from water and sunlight, two of the most abundant natural resources on Earth [10], offers a promising pathway for acquiring hydrogen. Solar water splitting transforms sunlight into hydrogen through photocatalytic, photoelectrochemical, and photovoltaic electrolysis routes [11,12,13]. The photocatalytic approach works without a corrosive electrolyte or external power, providing a more competitive pathway to hydrogen production.

The catalyst is the key to photocatalysis, and among various photocatalysts [14,15,16,17], titanium dioxide (TiO_2_)-based nanomaterials are the most widely used. TiO_2_ crystals were divided into anatase, rutile, and brookite phases [18,19,20], which have good photophysical properties and chemical stability, an appropriate band gap matching the redox level of water [21,22], and an adjustable microstructure regulated by hydrothermal, sol–gel, or redox methods [23,24]. Specifically, TiO_2_-related catalysts have wide applications in photocatalytic water decomposition and pollutant degradation [25]. The photocatalytic performance of TiO_2_ is affected by its microstructural characteristics. N-doping, photosensitizer modification, and composite strategy are important approaches to increasing the light absorption, carrier concentration, and photocatalytic activity of TiO_2_ crystals [26,27,28]. The final catalytic efficiency of different structures suggests microstructure-dependent catalytic activity. However, the wide band gap of TiO_2_ only responds to ultraviolet (UV) light (about 4% of the solar spectrum) instead of visible light (about 40% of the solar spectrum) [29,30,31]. Moreover, the harmful crystal defects and low specific surface area of TiO_2_ significantly hinder its photocatalytic performance [32,33]. Therefore, it is urgent to develop a nanocatalyst with a unique structure to enhance the photocatalytic performance of TiO_2_ in visible light. Although a variety of artificial TiO_2_ microstructures that mimic natural light-capturing behavior have been studied over the past decades, the precise control of TiO_2_ microstructures on the nanoscale in photocatalysis remains a major challenge.

Self-assembly is a common phenomenon in nature. For instance, self-assembly controls the chloroplast complex by programmatically regulating the size and aggregated spatial structure of functional motifs to drive natural photosynthesis. Moreover, self-assembly is a powerful strategy used to fabricate nanomaterials with unique microstructures and new functions [34,35,36,37]. Among numerous photoactive motifs, porphyrins are superior assembly modules with a rigid planar structure and strong visible light absorption capability [38,39,40]. However, they are hindered by the face recombination of photoelectrons and holes after irradiation and the risk of photobleaching without a protective external shell. The synergistic effect of porphyrin/TiO_2_ with microstructural regulation offers a promising strategy to address these issues.

**Figure 1:**
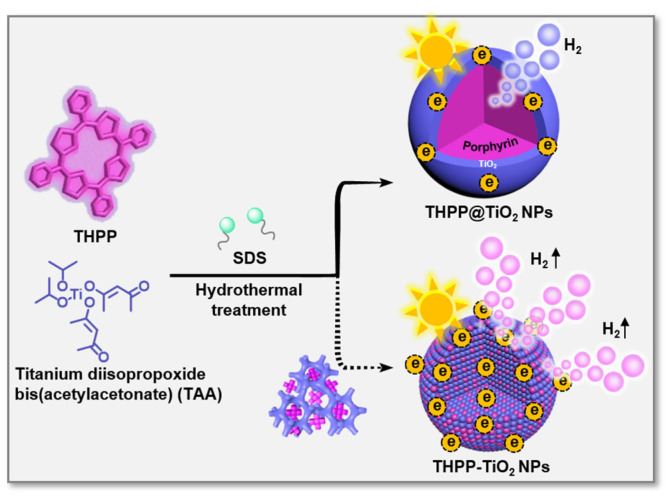
Schematic representation of the co-assembly process and two nanocomposites (THPP-TiO_2_ NPs and THPP@TiO_2_ NPs) with various microstructures. The blue and purple represent TiO_2_ and THPP species.

Herein, we developed two meso-tetra(4-hydroxyphenyl) porphyrin (THPP)/TiO_2_ nanocomposites with various assembled microstructures (Figure 1), i.e., hybrid-type nanoparticles (THPP-TiO_2_ NPs) and core@shell type (THPP@TiO_2_ NPs), through the tailored self-assembly of porphyrin and the hydrolysis/condensation of the TiO_2_ precursor, titanium diisopropoxide bis(acetylacetonate) (TAA). The controlled co-assembly can be regulated due to the stronger hydrogen bonding interactions between the hydroxyl group of the TiO_2_ scaffold and THPP than those of meso-tetra(4-benzenesulfonic acid)porphyrin (TSPP) and meso-tetra(4-carboxyphenyl)porphyrin (TCPP). The sodium dodecyl sulfate (SDS) surfactant and hydrothermal treatment further limited the size of the obtained nanocomposite and increased the crystallization degree of TiO_2_, respectively. Acting as a light-capturing antenna, porphyrin assisted the THPP-TiO_2_ NPs in harvesting and transferring visible light into a higher level of hydrogen using its large specific surface area, abundant active sites, superior light-capturing capability, and high crystallinity. Our advanced photocatalytic system provides a technically simple method for the microstructural regulation of catalysts to greatly improve photoenergy utilization and visible-light-responsive photocatalytic hydrogen evolution.

## 2. Results

### 2.1. The Optimization of Light-Capturing Blocks

**Figure 2:**
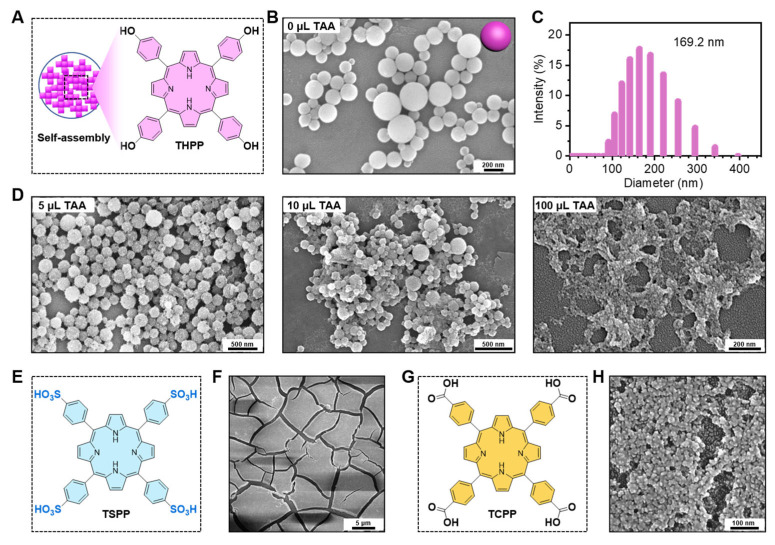
(**A**) Description of the self-assembly of THPP NPs; (**B**) scanning electron microscopy (SEM) image of THPP NPs; (**C**) diameter statistic of THPP NPs; (**D**) SEM images of THPP-TiO_2_ NPs prepared with 1 mg of THPP and various volumes of TAA; (**E**) chemical structure of TSPP; (**F**) SEM image of TSPP-TiO_2_ NPs; (**G**) chemical structure of TCPP; (**H**) SEM image of TCPP-TiO_2_ NPs.

The THPP building blocks were self-assembled into 169.2 nm smooth nanospheres in a 25 mL reaction system (Figure 2A–C). The smooth surface of the THPP NPs gradually became rough with the increasing ratio of TiO_2_ precursor (TAA). The proportion of TAA has a crucial role in the co-assembly process. The optimal ratio of TAA was determined according to the morphologies of the nanomaterials. The scanning electron microscopy (SEM) images of the THPP-TiO_2_ nanocomposites indicated that 5 μL of TAA could acquire a monodispersed and homogeneous distribution of TiO_2_ in nanoparticle than 10 μL or 100 μL (Figure 2D). The THPP in THPP-TiO_2_ NPs was replaced by TSPP (Figure 2E,F) or TCPP (Figure 2G,H) but both were unable to yield a homogeneous morphology similar to that of the THPP-TiO_2_ NPs. These results suggested that the benzene hydroxyl group in THPP had a stronger binding force with the hydroxyl group of TiO_2_ in the sol–gel preparation process, yielding a homogeneous incorporation.

### 2.2. The Regulation of Assembled Microstructure

**Figure 3:**
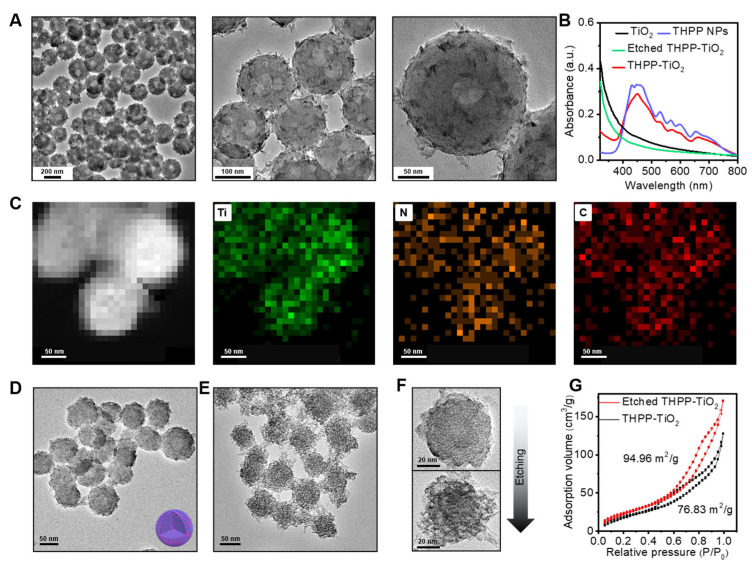
(**A**) Transmission electron microscopy (TEM) images of THPP-TiO_2_ NPs prepared without SDS at different magnifications; (**B**) UV–vis absorption spectra of the indicated samples; (**C**) element mapping of large-size THPP-TiO_2_ NPs; (**D**,**E**) TEM images of small-size THPP-TiO_2_ NPs before and after etching with NaOH (1 M), which was prepared assisted by SDS; (**F**) magnifying TEM images of THPP-TiO_2_ NPs before (top) and after (down) etching with NaOH; (**G**) N_2_ adsorption–desorption isotherms of small-size THPP-TiO_2_ NPs before and after etching.

The TEM images of THPP-TiO_2_ NPs revealed a rough morphology (Figure 3A). The UV–vis spectra indicated successful THPP loading (Figure 3B), which disappeared after etching by sodium hydroxide (NaOH). The elemental mapping further suggested a homogeneous distribution of Ti, N, and C in the nanoparticles (Figure 3C). To further decrease the nanoparticle size to improve the catalytic performance, we used an interfacial self-assembly microemulsion process driven by SDS micelle. As a result, the THPP-TiO_2_ NPs greatly decreased in size around the 50–100 nm range (Figure 3D). Moreover, the TEM images of the etched THPP-TiO_2_ NPs revealed an obvious pore structure after etching (Figure 3E,F). The specific surface area is an important factor of the catalyst in the photocatalysis process. The N_2_ adsorption–desorption isotherms were collected to detect the pore change. The results indicated that the specific surface area of the THPP-TiO_2_ NPs increased from 76.83 to 94.96 m^2^/g after etching (Figure 3G).

### 2.3. The Optimization for the Crystallinity of TiO_2_

**Figure 4:**
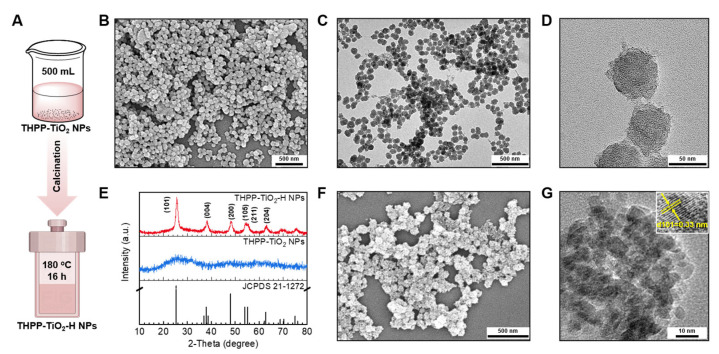
(**A**) Description of the scaled-up (500 mL) preparation and following hydrothermal treatment of THPP-TiO_2_ NPs; (**B**–**D**) SEM and TEM images of the obtained THPP-TiO_2_ NPs; (**E**) XRD patterns of THPP-TiO_2_ NPs, THPP-TiO_2_-H NPs, and the standard card (JCPDS 21-1272); (**F**,**G**) SEM and magnified TEM images of THPP-TiO_2_-H NPs (inset: detailed lattice fringe). The hydrothermal treatment is expressed as the -H suffix.

Anatase generates a higher oxidation capacity due to a higher band gap (3.2 eV), oxygen vacancies to capture electrons, and catalytic activity compared to those of rutile. Therefore, the amorphous TiO_2_ in THPP-TiO_2_ NPs were further changed to anatase-type TiO_2_ by hydrothermal treatment. To expand their application, the volume of the reaction system was increased from 25 to 500 mL (Figure 4A). The SEM and TEM images indicated that the products remained stable in size and morphology after scaled-up preparation (Figure 4B–D). The hydrolysis condensation product of TAA is amorphous TiO_2_ in THPP-TiO_2_, exhibiting no prominent peaks in the X-ray diffraction (XRD) pattern (Figure 4E) and changed to the TiO_2_ crystal of THPP-TiO_2_-H NPs after hydrothermal treatment. The characteristic peaks of THPP-TiO_2_-H NPs matched the JCPDS pattern (JCPDS 21-1272) of anatase. The SEM image of the THPP-TiO_2_-H NPs maintained a morphology similar to that before hydrothermal treatment (Figure 4F). The magnified TEM image indicated a 0.33 nm lattice spacing assigned to the (101) crystal face (Figure 4G, inset: lattice spacing) [41]. These results indicated that the THPP-TiO_2_-H NPs had superior anatase crystal structure.

### 2.4. Structure-Dependent Photocatalysis

**Figure 5:**
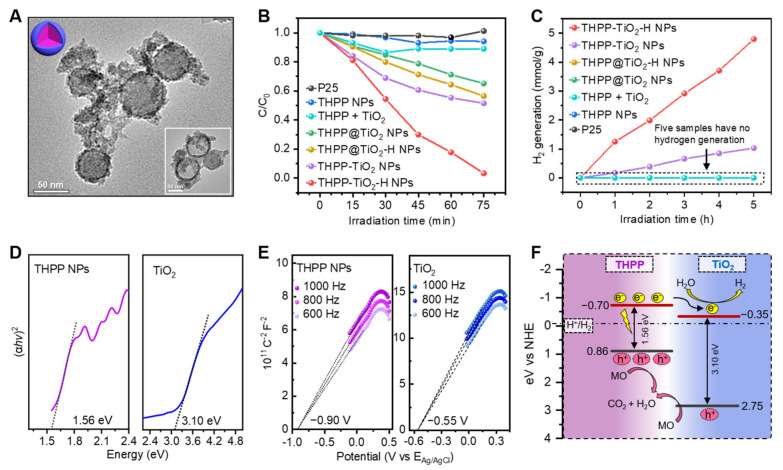
(**A**) TEM images of THPP@TiO_2_ nanospheres before and after (inset image) etching with NaOH (1 M); (**B**) relative degradation rate of MO by the indicated treatments. The mass of the catalysts was 6 mg in each catalytic system. The THPP + TiO_2_ is a mixture of THPP NPs (3.75 mg) of TiO_2_ NPs (2.25 mg). C_0_ is the initial concentration of MO; (**C**) photocatalytic hydrogen production of the indicated samples (2 mg) as a function of irradiation time. The THPP + TiO_2_ is the mixture of THPP NPs (1.25 mg) of TiO_2_ NPs (0.75 mg). The volume fraction of the triethanolamine sacrificial reagent (triethanolamine, TEOA) and the mass percent of the Pt co-catalyst were 10 vol % and 2%, respectively. (**D**) Tauc plots of (*αhv*)^2^ vs. E_g_; (**E**) Mott–Schottky plots of THPP NPs and TiO_2_ NPs; (**F**) description of photocatalytic mechanism in THPP-TiO_2_-H NPs.

Lastly, we investigated the photocatalytic performance and verified the catalytic mechanism of the nanocomposites. The hybrid microstructure of the THPP-TiO_2_ NPs was adjusted to the core@shell control samples (THPP@TiO_2_ NPs) through a two-step method, i.e., the self-assembly of THPP combined with a TiO_2_ shell coating. The TEM image of the etched THPP@TiO_2_ NPs showed a hollow TiO_2_ shell structure (inset, Figure 5A). Methyl orange (MO) is a representative pollutant often used to investigate the photocatalytic performance of catalysts. Compared to the negligible photocatalytic activity of the commercial titanium dioxide photocatalyst P25, the THPP-TiO_2_-H NPs revealed a higher degradation output, exceeding 96.7% after 75 min (Figure 5B). The photocatalytic hydrogen production capabilities of both THPP-TiO_2_-H NPs and THPP-TiO_2_ NPs indicated irradiated time-dependent hydrogen generation, respectively reaching 4.80 and 1.03 mmol/g, which was much higher than that of P25, THPP NPs, THPP + TiO_2_, THPP@TiO_2_-H NPs, and THPP@TiO_2_ NPs (Figure 5C). These results indicated that the commercial P25 control sample was unable to efficiently respond to visible light (>388 nm) [42,43] due to the intrinsic wide band gap (band gap 3.2 eV) of TiO_2_. Moreover, hybrid structure and hydrothermal treatment were essential in the exhibited photocatalytic activity, suggesting that the assembly could greatly increase the microstructure-dependent photocatalytic activity.

To further explore the photogenerated electron-transport process of catalysts in the photocatalytic process, the band energies of TiO_2_ and THPP NPs were investigated. According to the UV–Vis diffuse reflectance spectra, the energy gaps (E_g_) of the THPP assemblies and TiO_2_ NPs were calculated to be 1.56 and 3.10 eV (Figure 5D). The Mott–Schottky curve showed that the conduction band (E_CB_) positions of the THPP assemblies and TiO_2_ NPs were −0.70 and −0.35 eV vs. NHE (Figure 5E). According to the equation E_g_ = E_VB_ − E_CB_, the E_VB_ values of the THPP assemblies and TiO_2_ NPs were calculated to be 0.86 and 2.75 eV. The staggered band structures between THPP and TiO_2_ facilitate charge separation in the THPP-TiO_2_-H NPs. Under visible light irradiation, the electron–hole pairs are simultaneously generated at both THPP and TiO_2_. The photogenerated electrons at the conduction band of THPP would spontaneously transfer to the TiO_2_, with a lower conduction band potential. The transfer of the photogenerated carriers effectively accelerates the separation of photogenerated electron–hole pairs of THPP [44], yielding higher hydrogen production. Meanwhile, the surface Pt cocatalyst [38] facilitated photoelectron transport from TiO_2_ to H_2_O by inhibiting the undesired recombination of photo-generated electron–hole pairs on TiO_2_, improving the utilization efficiency of visible light. Additionally, the holes of THPP and TiO_2_ NPs were used for MO degradation (Figure 5F). The hybrid structure in THPP-TiO_2_-H NPs is beneficial to the above process. However, the core@shell structure in THPP@TiO_2_-H NPs is unable to efficiently transfer the electron to TiO_2_ due to the harmful recombination of electrons and holes, inducing low catalytic efficiency.

## 3. Materials and Methods

### 3.1. Materials

THPP, TSPP, TCPP, sodium dodecyl sulfate (SDS), and titanium diisopropoxide bis(acetylacetonate) (TAA, 75 wt.% in isopropanol) were purchased from Sigma-Aldrich (St. Louis, MO, USA). Sodium hydroxide (NaOH, 1 M) standard solution was obtained from Acros Organics (Geel, Belgium). Tetrahydrofuran (THF) was obtained from Tianjin Kermel Chemical Reagent Co., Ltd. (Tianjin, China). Commercial titanium dioxide (P25) was purchased from Degussa Co., Essen, Germany. All chemicals were used as received without further purification. X-ray powder diffraction (XRD) patterns were obtained using Bruker D8 Advanced (Bremen, Germany). The surface area of powders was measured using an ASAP2020 adsorption analyzer (Norcross, GA, USA). The photocatalytic system was purchased from PerfectLight Co., Ltd. (Beijing, China). The morphological observation was performed on the transmission electron microscopy (JEOL JEM-2010, Tokyo, Japan) operated at 200 kV accelerating voltage and scanning electron microscope (JEOL JSM-6710F). Ultraviolet and visible absorption spectroscopy was collected on a Xidi UV-5400 spectrophotometer (Shanghai, China).

### 3.2. Preparation of THPP Nanoparticles

The self-assembly method was used to prepare THPP NPs. Briefly, 1 mg of THPP powder was dissolved in 1 mL of tetrahydrofuran (THF) solvent under sonication for 5 min. Then, the mixture was injected into 25 mL of water and further grown at 25 °C for 24 h.

### 3.3. Preparation of THPP-TiO_2_ Nanocomposites

To decrease the size of the nanocomposites, the SDS surfactant was introduced into the above system. Briefly, 50 mg of THPP powder and 250 μL of TAA were dissolved in 50 mL of THF and stirred for 0.5 h at 25 °C. Subsequently, the solution was quickly added to 500 mL of SDS aqueous solution (0.01 M) under magnetic stirring, followed by magnetic stirring for 24 h. The TiO_2_ sample was prepared according to the same procedure but with the removal of THPP. The THPP-TiO_2_ NPs with large sizes were prepared according to the same procedure but lack of SDS. The pure TiO_2_ sample was prepared following the same procedure without adding THPP.

### 3.4. Preparation of THPP@TiO_2_ Nanocomposites

The THPP@TiO_2_ nanocomposites were prepared using a two-step method. Briefly, 1 mg of THPP power was dissolved in 1 mL of THF solvent under sonication for 5 min. The mixture was injected into 25 mL of SDS aqueous solution (0.01 M) and further grown at 25 °C for 24 h. Subsequently, 5 μL of TAA dispersed in 10 μL of THF solvent was added to the solution. The reaction system was allowed to further grow for 24 h and acquire the THPP@TiO_2_ NPs after centrifugation at 8000 rpm for 10 min and washing two times with water.

### 3.5. Hydrothermal Treatment

To increase the crystallinity, the TiO_2_ NPs, THPP@TiO_2_, and small-size THPP-TiO_2_ nanocomposites required a hydrothermal process. First, the resulting NPs solution was centrifuged at 8000 rpm for 10 min to remove excess reactants. Then, the precipitate was redispersed in water and transferred to a reactor containing a Teflon liner. Lastly, the reactors were sent to the oven and carried a hydrothermal treatment at 180 °C for 16 h. The ultimate products were collected using a centrifugation procedure at 8000 rpm for 10 min.

### 3.6. Photocatalytic Methyl Orange (MO) Decomposition

To test the photocatalytic performance, 6 mg of various nanocomposites or P25 were added to 3 mL of methyl orange (MO, 20 mg/L) dye aqueous solution, which was further stirred for 30 min in a dark environment. Then, the mixture was sent to the irradiation of visible light (PLS-SXE300/300UV, Beijing Perfectlight Technology Co., Ltd., 300 W, wavelength: UV cutoff filter > 400 nm). Subsequently, 400 μL of solution was taken out every 15 min and performed five times. These tested solutions were centrifuged at 8000 rpm for 10 min. The absorption of the supernatant was measured.

### 3.7. Photocatalytic Hydrogen Generation

The photocatalytic hydrogen generation assay was administrated in 50 mL of aqueous solution (pH = 8.1–8.2 adjusted with HCl) containing 5 mL of triethanolamine (TEOA, 10 vol%) sacrificial reagent and 41 μL of potassium chloroplatinate (5 mM). Then, 2 mg of tested samples were respectively added to various parallel solutions to harvest the hydrogen generation under visible light (UV cutoff filter > 400 nm) irradiation and tested using gas chromatography.

### 3.8. Mott–Schottky Tests

The Mott–Schottky plots and photocurrent response were measured on an electrochemical workstation (Autolab, Utrecht, The Netherlands) equipped with a standard three-electrode system. Briefly, the electrolyte solution is Na_2_SO_4_ (0.5 M) aqueous solution, which was purged with N_2_ to remove air before utilization. The working electrode was prepared by coating the catalysts onto the Pt/C electrode. The reference and counter electrode respectively adopted the Ag/AgCl electrode and platinum plate.

## 4. Conclusions

In this paper, we controllably established two microstructural regulation modes to explore the structure-dependent photocatalytic performance. In the co-assembly process, the benzene hydroxyl group acquired optimal morphology and hybrid uniformity via the strong interaction between the hydroxy groups of THPP and TiO_2_. The introduction of the SDS template efficiently limited the growth of the resulting nanocomposites even in large-scale applications. Compared to the core@shell structure of the THPP@TiO_2_ nanocomposites, the THPP-TiO_2_ nanocomposites with hybrid microstructure exhibited the close contact of porphyrin with TiO_2_ and abundant catalytic sites, which promoted photogenerated electron–hole separation. Anatase TiO_2_ accelerated the transport efficiency of photoelectrons to Pt on the surface, leading to high MO decomposition efficiency and photocatalytic hydrogen production. The ingenious assembly of structure-regulated nanocatalysts provides insight into the fabrication of advanced photocatalysts and photocatalytic activity modulation.

## Data Availability

The original contributions presented in this study are included in the article; further inquiries can be directed to the corresponding author.
